# Targeting the gut-pancreatic axis: microbial modulation of immunotherapy in pancreatic cancer

**DOI:** 10.3389/fimmu.2026.1682390

**Published:** 2026-02-02

**Authors:** YuRong Yuan, Wei Zhang, Deqiang Wang

**Affiliations:** 1 Jiangsu University, Affiliated Hospital of Jiangsu University, Zhenjiang, China; 2 Department of Gastroenterology, Affiliated Hospital of Jiangsu University & Institute of Digestive Diseases, Jiangsu University, Zhenjiang, China; 3 Department of Oncology, Digestive Disease Institute & Cancer Institute of Jiangsu University, Affiliated Hospital of Jiangsu University, Zhenjiang, China

**Keywords:** gut microbiota, gut-pancreatic axis, immunotherapy, pancreatic ductal adenocarcinoma, tumor microenvironment

## Abstract

With advances in microbial sequencing technology, the role of microorganisms in cancer development and treatment has been increasingly explored. The gut microbiota, as a key shaper of both innate and adaptive immunity, is believed to migrate from the gut and colonize the pancreas, thereby influencing the tumor microenvironment(TME) of pancreatic cancer. Pancreatic cancer exhibits treatment resistance due to its immunosuppressive TME and high interstitial density. Multiple sequencing analyses of pancreatic tumor tissues have revealed that alterations in the tumor-associated microbiota are associated with prognosis and treatment response, suggesting that the microbiota may serve as a complementary modality in immunotherapy. This paper describes potential pathways by which the gut microbiota can migrate to the pancreas and analyzes changes in tumor microbiota composition. It also identifies microbiota types associated with prognosis, and summarizes treatment strategies leveraging the gut-pancreas axis to enhance the personalization and precision of care. It critically examines the limitations of existing research, and aims to leverage microbiome ecology to overcome the immune -suppressive barrier in pancreatic cancer and improve patient outcomes.

## 1 Introduction

Pancreatic cancer is a highly aggressive cancer with a significant burden, expected to become the second leading cause of cancer-related deaths in the United States by 2030 (1). Pancreatic ductal adenocarcinoma (PDAC) is the most common type of pancreatic solid tumor, accounting for over 95% of all pancreatic cancer (1). Nonspecific clinical symptoms, early micro-metastasis, and a highly fibrotic, immunosuppressive TME make treatment of PDAC challenging (1, 2). These factors together lead to a five-year survival rate of only 13%, making it one of the deadliest cancers (3).

Facing this significant challenge, current treatment approaches focus on surgical removal supported by systemic chemotherapy. For patients with good performance status, the FOLFIRINOX regimen (oxaliplatin, irinotecan, fluorouracil, and calcium folinate) or albumin-bound paclitaxel with gemcitabine is now a standard first-line treatment option (1). Although these approaches significantly improve patient survival, their overall effectiveness remains limited and frequently associated with drug resistance and high rates of recurrence (4–6). Despite the rise of precision medicine, where targeted therapies for specific genetic mutations (e.g., *BRCA, KRAS*) and immune checkpoint inhibitors (ICIs) offer hope for a small group of patients, response rates to these new treatments remain very low for most of PDAC patients (7–10). Consequently, developing innovative adjuvant strategies to address treatment resistance and enhance patient outcomes has become a critical challenge in current clinical practice.

Recent evidence suggests that gut microbiota and its translocation to the PDAC TME influence its progression and treatment response, indicating a new potential avenue for therapy. The main mechanism of gut microbiota translocation is due to disruption of the intestinal barrier and dysbiosis. In PDAC or its precancerous stages, various risk factors, such as smoking, alcohol abuse, and chemotherapy can damage the intestinal mucosal, mechanical barrier, reduce tight junction protein levels, and worsen mucosal injury through an increase in harmful bacteria (11–13). This process enables bacteria and their metabolites, which are normally symbiotic in the gut, to breach the intestinal mucosa and access the portal venous circulation and lymphatic system. Once translocated to the pancreas, these microbiome components can trigger both local and systemic immune responses, leading to alterations in the TME (14, 15).

In this article, we first explore the potential origins of pancreatic microbiome from the gut. We then review and analyze the types of microorganisms linked to prognosis in PDAC, examining possible mechanisms by which microbes affect immunotherapy. Finally, we look ahead to the potential of innovative microbiome-based therapeutic strategies for improving PDAC immunotherapy.

## 2 Microbial translocation from the intestine to the pancreas: sources and compositional changes

### 2.1 Intestinal origin of microorganisms in the pancreas

**Figure 1:**
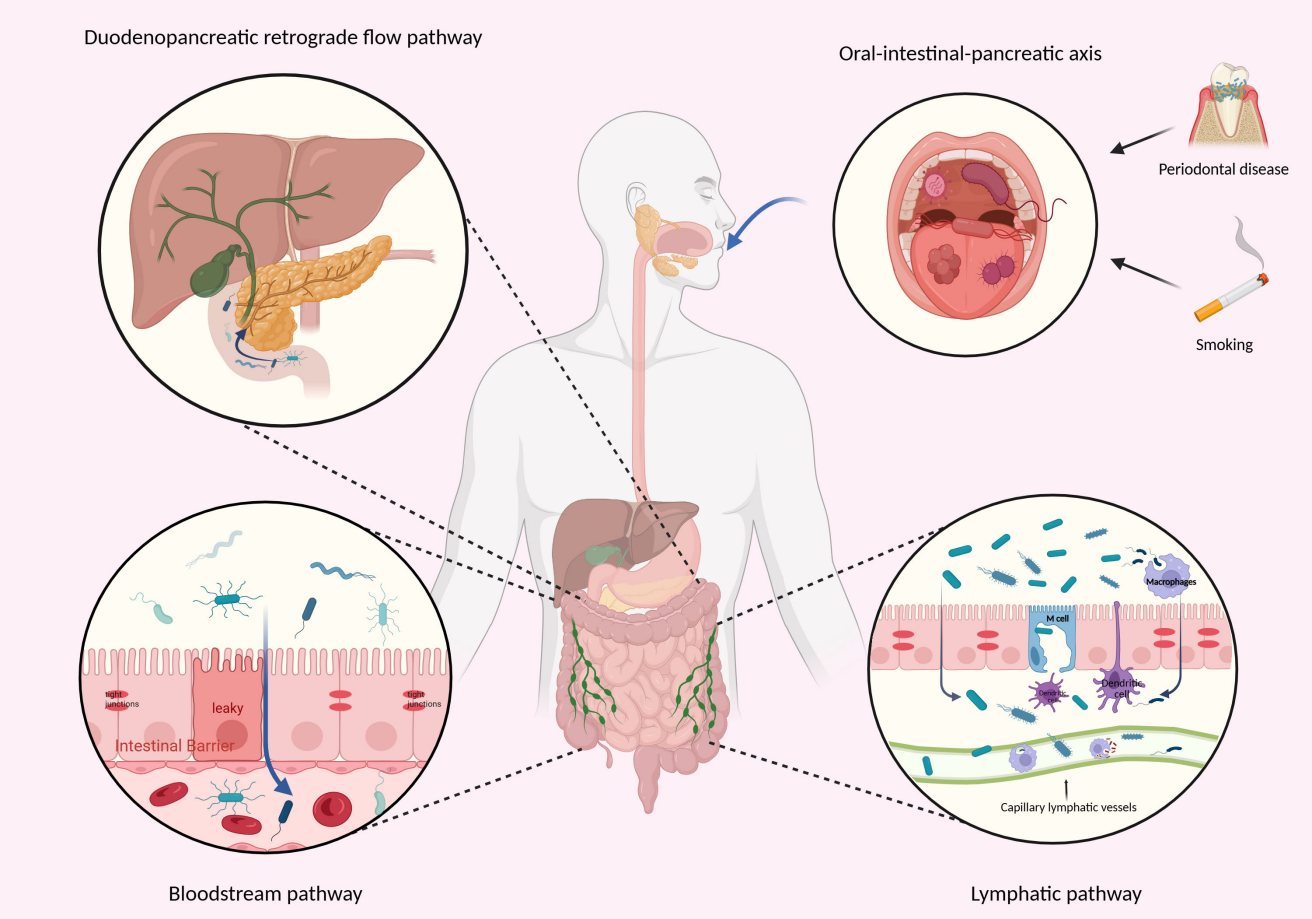
Possible pathways for pancreatic microbial translocation include the Duodenopancreatic retrograde flow pathway, where intestinal bacteria enter the pancreas via retrograde migration through the pancreatic duct; the Bloodstream pathway, where bacteria enter the bloodstream following intestinal barrier disruption and migrate to the pancreas; and the lymphatic pathway, where bacteria drain to the pancreas via MLNs or migrate after phagocytes capture intestinal bacteria. Additionally, it also includes the potential oral-intestinal-pancreatic transfer axis caused by smoking and periodontal disease. Created in BioRender. Yuan, Y. (2026) https://BioRender.com/gxjwbbf.

Under normal physiological conditions, several mechanisms work together to maintain a low biomass environment in the pancreas. These include the antimicrobial effects of pancreatic enzymes, the mechanical barrier provided by the sphincter of Oddi, the integrity of the intestinal mucosal barrier, and the immune filtration role of the liver (16). Microbial translocation relies on the disruption of these barriers (**Figure 1**).

Evidence clearly shows a distinct, active microbial community in pancreatic cancer, with much higher abundance than in normal pancreatic tissue. Its microbial profile suggests it mainly comes from the gut. Ghaddar found gastrointestinal pathogens like *Campylobacter* spp*, Fusobacterium nucleatum, Leptotrichia* spp*, and Clostridioides difficile* enriched in pancreatic tumors through single-cell analysis of PDAC groups (17). Pushalkar demonstrated gut microbiota translocation to the pancreas by detecting orally given fluorescent *Enterococcus faecalis* and GFP-labeled *Escherichia coli* in pancreatic tissues of wild type(WT) mice. They also found a bacterial peak at just 0.5 hours after ingestion, indicating a swift retrograde flow of gut bacteria through the pancreatic duct into the pancreas (18). Aykut’s research similarly observed this rapid translocation in both WT mice and pancreatic cancer mouse models, which used oral force-feeding, with GFP-labeled *Saccharomyces cerevisiae (*19). Mouse models demonstrate the potential for retrograde microbial migration through the pancreatic duct via the duodenal papilla, supported by physiological and pathological evidence. While Nejman showed through human pan-cancer microbiome analysis that the Proteobacteria-dominated microbiome of pancreatic cancer mirrors that of the duodenum, direct evidence from human samples has yet to be established (20).

Similarly, with oral gavage, Ma tracked the live-cell tracer calcein-AM and detected the periodontal pathogen *Porphyromonas gingivalis* in the pancreas and feces of mice, supporting a potential oral-intestinal-pancreatic translocation route (21, 22).

Increased permeability of intestinal barrier, resulting in intestinal permeability, is often the initial step for microbial translocation to various organs and the activation of inflammatory cascades (23–25). Pancreatitis and risk factors linked to pancreatic cancer, such as obesity and smoking, are often associated with increased intestinal permeability and gut microbiota dysbiosis (26). Early studies with mouse models of induced pancreatitis, along with blood endotoxin detection and electron microscopy of mucosal ultrastructural changes, indicated that intestinal bacteria enter the bloodstream by compromising tight junctions in the intestinal mucosa (27). Study in feline models of acute pancreatitis also indicate that *Escherichia coli* can spread to the pancreas through the bloodstream and colon wall, as well as reflux into the pancreatic duct (28).

The gut microbiome and its metabolites play a key role in shaping immunity. Phagocytes usually capture these components to present antigenic information to T cells, which helps regulate future immune responses. A study using single-strain germ-free mice representing dominant gut bacterial phyla, including *Bacteroidetes, Firmicutes, Proteobacteria, Actinobacteria, and Fusobacteria*, found that the vast majority (88%) of gut microbes could migrate to and survive in proximal lymph nodes, while nearly half (47%) could survive in systemic lymphoid organs such as distal subcutaneous lymph nodes tissue (29). An earlier study showed that in Myeloid differentiation primary response 88 (Myd88)-deficient mice or under dysbiosis conditions, non-invasive *Salmonella* is transported to the mesenteric lymph nodes (MLNs) by CX3CR1^hi^ mononuclear phagocytes through a CCR7-dependent process, leading to T cell responses and IgA production (30). Surprisingly, exogenous immunotherapy—ICIs—has been shown to cause intestinal bacterial translocation to secondary lymphoid organs, displaying a dynamic translocation pattern characterized by an initial dominance of *Enterococcus faecalis* followed by a later dominance of *Lactobacillus johnsonii (*31). The selection, capture, and transport of bacteria from the intestine to the MLN may enable intestinal microbiota to reach the pancreas via the lymphatic drainage pathway. Recent studies have also used real-time *in vivo* tracing technology to detect a significant increase in bacterial signals within phagocytes in the liver and Peyer’s patches of lymph nodes in obese mouse models, showing gut microbiota translocation via the circulatory system and liver tissue (32).

Experimental evidence indicates multiple potential pathways for gut-to-pancreas microbial translocation, with core mechanisms and corresponding animal models summarized below (**Table 1**). These pathways provide a structural basis for gut microbiota-mediated regulation of the pancreatic TME and immunotherapeutic response.

**Table 1:** Primary pathways of microbial translocation (ductal, vascular, lymphatic, phagocytosis-mediated) and associated animal models.

Translocation Pathway	Animal Model	Detected Microorganisms/Tracers	Core Mechanism	Reference
Duodenopancreatic retrograde flow pathway	Wild type mice	Fluorescently-labeled *Enterococcus faecalis*, GFP-labeled *Escherichia coli*	Intestinal bacteria flow retrograde through the pancreatic duct and rapidly colonize the pancreas	(18)
	Wild type mice,LSL-KrasG12D mice	GFP-labeled *Saccharomyces cerevisiae*		(19)
	C57BL/6 mice	Calcein AM-labeled *P. gingivalis*		(22)
Bloodstream pathway	Severe acute pancreatitis rats	Plasma endotoxin level	Disruption of tight junctions in the intestinal mucosa allows bacteria to penetrate the mucosa and enter the portal venous circulation, subsequently spreading hematogenously to the pancreas.	(27)
	Severe acute pancreatitis rats	*Escherichia coli*		(28)
Lymphatic Pathway	GF mice with single microbial	Fifty-three bacterial species covering the 5 dominant phyla: *Bacteroidetes, Firmicutes, Proteobacteria, Actinobacteria*, and *Fusobacteria*	Bacteria enter the lymphatic circulation via mesenteric lymphatic vessels and are transported to the pancreas through secondary lymphoid organs.	(29)
	Myd88^-^/^-^ mice and Myd88^+^/^-^ littermate controls,CCR7^-^/^-^ mice and CCR7^+^/^+^ littermates,CX_3_CR1-GFP mice	Non-invasive *Salmonella*	Under dysbiosis or MyD88 deficiency, CX_3_CR1^hi mononuclear phagocytes-mediated bacterial translocation to MLN	(30)
	Mouse model of melanoma	Rich translocated bacteria such as *Enterococcus* spp, *Lactobacillus johnsonii*, and *Enterobacteriaceae*	ICIs induces lymph node remodeling and dendritic cell activation, promoting the selective migration of specific subpopulations of gut bacteria to extraintestinal tissues.	(31)
	High-fat diet-induced obese mouse model, leptin production genetic obese model, leptin receptors genetic obese model	TetraAA-BHQ fluorogenic probe (containing TAMRA fluorophore and BHQ-2 quencher), BODIPY-tagged lipid (lymphatic vessel tracer), Hoechst 33342 (nuclear staining), Alexa Fluor 647-labeled anti-F4/80 antibody (Kupffer cell staining)	Intestinal barrier disruption enables microbial translocation via the blood and lymphatic systems, with detectable fluorescent signals in intestinal mucosal capillaries, subcutaneous vessels and the liver.	(32)

### 2.2 Changes in the microbial composition of tumors

The number and diversity of genes in the human microbiome significantly surpass those in the human genome (33), and dysbiosis of the microbiome has been linked to various malignant tumors (34, 35). Ley et al. propose that the microbial diversity in the human gut results from co-evolution between microbial communities and their hosts, and that the human environment applies selective pressure on microbial colonization (36). Merali’s team systematically described and summarized PDAC biomarkers from various microbial enrichment chamber in the human body, discovering that each microbial enrichment chamber has its own unique profile (37). This suggests that, based on intestinal microbial dysbiosis, during translocation and colonization, microbial communities change further due to environmental pressures and the microorganisms’ inherent tendencies and preferences. This emphasizes the importance of assessing the microbial composition within pancreatic tumors to better understand the development of pancreatic cancer and how it responds to treatment.

As early as 2017, Geller et al. performed a quantitative analysis of bacterial 16S rRNA in surgical resection samples of PDAC and normal pancreatic control samples, discovering that the bacterial DNA in pancreatic cancer samples was significantly higher than that in normal controls (76% vs. 15%, P < 0.005). Subsequent Fluorescence *in situ* hybridization and immunohistochemistry studies also verified the presence of tumor-associated bacteria in human PDAC samples. To identify the types of tumor-associated bacteria, deep sequencing of bacterial 16S rDNA amplified by PCR was conducted on 65 samples, showing that the most common bacteria in pancreatic cancer tissue belonged to the *Enterobacteriaceae* and *Pseudomonadaceae* families within the *Gammaproteobacteria* class (38). Subsequently, Pushalkar et al. discovered that pancreatic cancer shows enrichment of 13 bacterial phyla, mainly *Proteobacteria* (45%), *Bacteroidetes* (31%), and *Firmicutes* (22%) (18). These three phyla have been consistently enriched across multiple studies on tumor-associated microbiota in pancreatic cancer, with *Proteobacteria* exhibiting the highest consistency (39–46). Thus, *Proteobacteria* are the fundamental phylum in the microbiota of pancreatic cancer tumors. Moreover, it exhibits a dynamic profile of sustained enrichment throughout the entire spectrum from chronic pancreatitis to advanced pancreatic cancer (46). Following the gradual discovery of the role of bacterial communities, the potential of fungi in pancreatic cancer has also begun to emerge. *Malassezia* are also significantly enriched in PDAC tumor tissues and promote tumor progression (19). Subsequently, pan-cancer fungal analysis further confirmed that the fungal burden in pancreatic cancer tissues was not only significantly increased and tumor-specific, but also primarily colonized within cancer cells. This provides crucial evidence for the direct involvement of fungi in tumor cell behavior (47, 48). These studies collectively outline a preliminary map of the PDAC tumor-associated microbiome: dominated by the *Proteobacteria* phylum and characterized by a significant enrichment of fungal communities, including *Malassezia*.

Having established enrichment of gut-derived microbiota within tumors, the research focus naturally shifts to its association with clinical prognosis naturally. Riquelme et al. discovered that tumor-associated microbiome features (*Pseudoxanthomonas*-*Streptomyces*-*Saccharopolyspora*-*Bacillus clausii*) can strongly predict long-term survival (LTS) rates (49). Studying the differences in microbiome composition between short-term survival (STS) patients and LTS patients is seen as a more effective way to predict prognosis and how patients will respond to treatment (39, 40, 46, 49–54). (**Table 2** reports microbial features linked to survival in pancreatic cancer). However, the same bacterial genus can have opposite prognostic values in the same study or across different studies. For the *Proteobacteria* phylum, which showed the highest enrichment consistency in the study, different genera within this phylum demonstrated opposite prognostic values. *Pseudomonas*, a relatively common genus in tumor tissues, was associated with longer survival in the study by Gao (51). *In vitro* experiments showed that *Pseudomonas fluorescens* inhibits pancreatic cancer cell proliferation and induces apoptosis, demonstrating notable antitumor activity (51). In contrast, the report by Guo indicates that *Pseudomonas* was more prevalent in aggressive basal-like tumors and linked to worse prognosis, potentially playing a role in inflammation and contributing to pancreatic carcinogenesis (55). This apparent paradox profoundly reveals the complexity of tumor microbiome research, whose prognostic value is neither straightforward nor fixed. This divergence likely arises from several key factors: First, tumor molecular diversity is crucial. Guo’s study focused on the basal-like subtype, a specific high-risk group whose unique immunosuppressive and inflammatory TME may influence microbial function, shifting them from harmless commensals or bystanders into accomplices of oncogenic agents (55). In contrast, Gao’s study did not stratify by subtype, and its results may reflect the overall effect across mixed tumor subtypes (51). Second, strain-level specificity must not be overlooked. The genus *Pseudomonas* comprises diverse species with markedly different pathogenic and probiotic potential. A simplistic genus-level analysis risks hiding critical distinctions. Additionally, differences in research methods and TME contexts, such as the functional insights provided by metagenomics versus the community structure shown by 16S rRNA sequencing, alongside variations in patient genetics, geographic factors, and tumor immune states, collectively influence the final phenotypic outcome of the microbiome. *Akkermansia muciniphila* shows a similar bidirectional effect. In a study by May, *Akkermansia muciniphila* was a characteristic of the STS cohort in patients receiving gemcitabine chemotherapy (52), which contrasts with its previous studies reporting it as a probiotic that improves intestinal barrier function and has anti-inflammatory properties (56). This suggests it may play a pro-carcinogenic role under certain conditions. This paradox could arise from the TME reshaping microbial functions. For example, *Akkermansia muciniphila*’s ability to degrade mucin benefits the gut but may also disrupt tissue barriers or activate carcinogenic pathways in the pancreas. Additionally, strain specificity, host factors, and the overall composition of the microbial community can all influence its ultimate effect. Therefore, as with *Pseudomonas* species, the prognostic value of *Akkermansia muciniphila* is not straightforward, underscoring the importance of understanding microbial functions in specific contexts. Future studies should move beyond simple “microbiome-prognosis” correlations and focus on elucidating microbial functions at the strain level. This involves integrating metagenomics with culture-based microbiome research in the context of specific tumor subtypes and the spatial dynamics of the TME.

**Table 2:** Microbial characteristics associated with survival in pancreatic cancer.

Sample Collection	Sample Site	Geographic Region	Methodology	Treatment Status (untreated or pre-treated)	Microbiome Technique	Long Survival-related Signatures	Short Survival-related Signatures	Reference
FFPE samples	Pancreatic tumor tissue	USA	MD Anderson Cancer Center (MDACC): LTS (n=22), STS(n=21) Johns Hopkins Hospital (JHH): LTS (n=15), STS (n=10)	MDACC: (LTS:17 pre-treated,4 untreated),(STS: 14 pre-treated,8 untreated) JHH: not mentioned	16S rRNA gene sequencing	Class level: *Alphaprotebacteria*, *Sphingobacteria, Flavobacteria.* Genus level: *Saccharopolyspora, Pseudoxanthomonas, Streptomyces, Bacillus Clausii*	Class level: *Clostridia, Bacteroidea*	(49)
FFPE samples	Pancreatic tumor tissue	Japan	LST (n=27), STS (n=25)	10 untreated,42 pre-treated	16S rRNA gene sequencing		*Bacteroides, Lactobacillus, Peptoniphilus*	(39)
FFPE samples	Pancreatic tumor tissue	China	LTS (n=29), STS (n=76)	Not mentioned	16S rRNA gene sequencing	*Bacillus*	*Exiguobacterium*	(40)
FFPE samples	Pancreatic tumor tissue	China	LTS (n=17), STS (n=13)	untreated	16S rRNA gene sequencing	*Sphingomonas, Megasphaera, Bradyrhizobium, hgcI_clade, Desulfovibrio, Flavobacterium, Enhydrobacter, and Megamonas*	*Clostridium sensu stricto 1, Actinomyces, Porphyromonas, Aggregatibacter, and Neisseria*	(50)
FFPE samples	Pancreatic tumor tissue	Netherlands	LTS (n=12),STS (n=13)	untreated	Metatranscriptomic analysis	*Comamonas, Roseomonas, and Sphingobacterium*	*Sulfurivermis, Thauera, Sulfuritortus, Sphingopyxis, Novosphingobium, Acidovorax, Capnocytophaga*	(53)
Frozen samples	Pancreatic tumor tissue	USA	LTS (n=9),STS (n=9)	pre-treated	16S rRNA gene sequencing	*Arthrobacter* sp.*, Corynebacterium tuberculostearicum, Serratia marcescens, and Mycobacterium* sp.	*Akkermansia muciniphila, Streptococcus* sp. *And Lachnospiraceae NK4A136 group*	(52)
Frozen samples	Pancreatic tumor tissue	China	Patients rich in *Ralstonia pickettii* _ B(n=69), Patients lacking *Ralstonia pickettii _ B*(n=29)	Not mentioned	2bRAD-M sequencing		*Ralstonia pickettii_B*	(54)
Frozen samples	Pancreatic tumor tissue	China	Tumor and paired paracancerous tissue (n=53)	untreated	16S rDNA sequencing	*Pseudomonas*		(51)
fresh-­frozen Endoscopic Ultrasound-Fine Needle Aspiration (EUS-FNA) samples	Pancreatic tumor tissue	China	LTS (n=28), STS (n=35)	untreated	16S rRNA amplicon sequencing	*Fermentimonas caenicola, Skermanella aerolata, Limosilactobacillus reuter*	*Eggerthia catenaformis and Cardiobacterium hominis*	(46)

Notably, sampling and sequencing strategies directly influence the accuracy and resolution of taxonomic detection. Standard sampling methods include surgical resection, endoscopic ultrasound-guided fine needle aspiration (EUS-FNA), and bile collection. Surgical resection offers intact tumor tissue, although surgical manipulation may change microbial distribution (57). EUS-FNA is minimally invasive but yields limited sample volume (58). Bile samples reflect the biliary tract microbiota, yet their direct relevance to pancreatic tumors requires further validation (59). Formalin-fixed paraffin-embedded (FFPE) samples are the mainstay of retrospective clinical studies, but DNA degradation during embedding and storage impairs the sensitivity of microbial detection, especially in low-microbial abundance environments (52). Fresh frozen samples (e.g., surgical resections, EUS-FNA) maximize DNA preservation and thus represent a superior alternative to FFPE specimens (46, 52).

Low biomass in pancreatic tumor tissues is a well-documented challenge. The poor reproducibility of tumor-specific microbiome studies underscores the urgency of optimizing DNA/RNA extraction protocols and standardizing the analysis of sequencing data (60, 61). 16S rRNA sequencing remains the main method, reliably identifying taxa at the genus level but lacking enough resolution at the species level. This limitation significantly contributes to the conflicting prognostic associations of genus-level microbiota across studies (50, 55). Metagenomic sequencing has enhanced detection resolution, but cross-validation with tumor tissue samples from various sources remains crucial to ensure the specificity and accuracy of taxonomic assignments (62).

## 3 Potential mechanisms by which the microbiome influences immunotherapy

Having established the presence of gut- and tumor-associated microbiomes and their clinical significance, current research focuses on how these microbiomes influence the tumor immune microenvironment (TME). Through their metabolites, cellular components, or direct immune interactions, the microbiota significantly shapes the immune landscape of pancreatic cancer. It also impacts its response to treatment across various levels, including innate immunity, adaptive immunity, and physicochemical barriers.

### 3.1 Regulation of innate immune cells

The innate immune system acts as the body’s first line of defense against tumors. Thomas et al. observed increased infiltration of CD45^+^ immune cells in PDAC xenograft mice following microbial depletion (63). This finding in a preclinical PDAC model, consistent with evidence from other tumor preclinical models, suggests that the microbiota may shape the TME by regulating the recruitment and function of innate immune cells (64, 65).

The microbiome associated with pancreatic cancer differentially activates Toll-like receptors (TLRs), particularly TLR2, TLR4, and TLR5, via its components, such as lipopolysaccharide(LPS) and flagellin (18). In the PDAC model, this activation induces tumor-associated macrophages (TAMs) to polarize toward an immunosuppressive M2-like state via the TLR-TRAF6 signaling pathway (18). Specifically, this is characterized by decreased expression of co-stimulatory molecules such as MHC II and CD86, along with increased production of the anti-inflammatory cytokine IL-10 (18). In another *in vivo* and *in vitro* experiment using a pancreatic cancer model, sustained activation of the TLR4/NF-κB signaling pathway under LPS stimulation was similarly observed, leading to an increased proportion of M2-like macrophages within the TME (66). Concurrently, analysis of the PDAC database shows that prolonged LPS exposure increases Programmed Death-Ligand 1 (PD-L1) expression in tumor cells, thereby reducing the efficacy of ICIs (66). Macrophages treated with PDAC gut microbiota extracts exhibited reduced expression of CD44 and PD-1 in both CD4^+^ and CD8^+^ T cells (18). Similarly, *Malassezia* enriched in pancreatic cancer tumors exhibits cell wall sugar structures that are recognized by mannose-binding lectin (MBL), thereby activating the MBL–C3 complement cascade (19). This mechanism was directly validated in PDAC mouse model, large amounts of activated C3a bind to the C3aR receptor on the surface of pancreatic cancer cells, thereby directly promoting tumor proliferation (19). Beyond the complement cascade, *in vitro* study shows that β-glucan structures from PDAC tumor-enriched fungi (*Alternaria* and *Malassezia*) are recognized by Dectin-1 receptors on PDAC cancer cell surfaces, thereby activating downstream Src–Syk–CARD9–NFκB signaling, promoting IL-33 secretion (67). Subsequently, vivo studies in a PDAC mouse model confirmed the ability of specific fungi to promote IL-33 secretion by PDAC cells, as well as the critical role of IL-33 secretion in recruiting and activating Th2 cells, ILC2 cells, and Treg cells within the TME (67). Notably, under inflammatory and other tumor models, mechanisms involving biochemical factors such as reactive oxygen species (ROS) and oxidative stress have also been found to promote the extracellular secretion of IL-33 (68, 69). Moreover, in the context of pancreatic cancer, IL-33 simultaneously exhibits other tumor-promoting mechanisms distinct from those induced by fungi (70, 71). Another study under the PDAC model demonstrated that IL-33-stimulated ILC2 cells derived from myeloid cells possess antitumor immune potential by recruiting CD103^+^ DCs and activating CD8^+^ T cells (72). It also suggests that dual antitumor effects can be achieved by blocking PD-1 expression on ILC2 cells and supplementing IL-33 (72). The differing outcomes of the IL-33-ILC2 axis under distinct immune TME suggest that the impact of microbial-mediated innate immune cell activation pathways requires investigation and discussion across various tumor types, locations, and tumor immune potentials. Similar to their tumor-suppressing effects in most cancers (73, 74), the probiotic combination of *Lactobacillus casei* and *Lactobacillus reuteri* promotes M1 polarization of macrophages by inhibiting the TLR4-MyD88 signaling pathway in pancreatic cancer cells, thereby enhancing antitumor immune responses (75). Additionally, analysis using pancreatic cancer mouse models and metabolomics showed that this probiotic combination can decrease intestinal *Alloprevotella* abundance and increase azelate (75).

Concurrently, the microbiome promotes the accumulation of myeloid-derived suppressor cells (MDSCs) within the PDAC TME (18). After antibiotics eradicated the microbiome, MDSCs infiltration dropped significantly, showing that the microbiome plays a crucial role in maintaining the immunosuppressive myeloid cell population in pancreatic cancer (18). Research indicates that *Fusobacterium nucleatum*, driven by Fap2 to colonize pancreatic cancer cells and normal pancreatic epithelial cells, specifically induces the release of chemokines CXCL1, IL-8, GM-CSF, and MIP-3α (76). These factors promote the proliferation and migration of pancreatic cancer cells and the malignant transformation of normal cells through both autocrine and paracrine mechanisms (76). Similarly, through the CXCL1–CXCR2 axis, it promotes pancreatic cancer cell migration and invasion via autocrine signaling and attracts MDSCs infiltration via paracrine signaling to suppress CD8^+^ T cells, thereby creating an immunosuppressive TME (77).

Neutrophils are the most rapidly responding innate immune cells (78). *Porphyromonas gingivalis* enriched in pancreatic tumors significantly recruits tumor-associated neutrophils by upregulating chemokines (22). This process, accompanied by elevated neutrophil elastase, creates a pro-inflammatory and immunosuppressive TME that promotes tumor growth while suppressing the function of CD8^+^ T cells (22). In contrast, microbiome analysis based on a cohort of Chinese PDAC patients revealed that a lower Eubacterium/Bacteroides ratio in tumors and peri-tumor tissues predicted better prognosis in PDAC patients (40). Subsequently, in a PDAC mouse model, oral administration of the probiotic *Bacillus coagulans* demonstrated significant PDAC tumor suppression accompanied by substantial infiltration of inflammatory neutrophils in the TME (40). However, the detailed molecular pathways by which *Bacillus coagulans* modulates neutrophil function require further investigation. Notably, *Bacillus coagulans* has also been reported to exert immunomodulatory effects in other tumor models, though its cellular targets and mechanisms may vary across tumor types (79, 80).

In the PDAC TME, natural killer (NK) cells show impaired local infiltration and phenotypic imbalance. Their infiltration rate is very low (making up only 3.5% of tumor-infiltrating lymphocytes), and their phenotype is more immature (81, 82). Tumor cells can also evade NK cell surveillance by upregulating ligands that cause downregulation of NK cell activity receptors (82). Worse still, under the PDAC immunosuppressive TME, NK cells produced large amounts of the immunosuppressive cytokine IL-10, showing a shift toward a regulatory phenotype (81). Yu et al. found that microbe-associated soluble components under PDAC conditions exert multiple inhibitory effects on NK cells, including reduced cytotoxicity, migration, IFNγ secretion, and perforin expression, thereby contributing to the regulatory remodeling of NK cells (83). Unlike tumor-associated microbial effects, a recent study shows that in PDAC mouse models, the probiotic *Lactobacillus reuteri* improves antitumor activity by increasing NK cell infiltration and function (84). This antitumor effect has been shown to depend on NK cell-mediated innate immunity, although its specific mechanism requires further investigation.

The mentioned tumor-associated microbes have shown potential to promote the development of an immunosuppressive TME in studies exploring mechanisms that regulate innate immunity. Conversely, antibiotic clearance and probiotic supplementation demonstrate significant antitumor potential, indicating that manipulating the microbiome under tumor conditions could be promising for boosting immunity and working together with other immunotherapies.

### 3.2 Regulation of adaptive immune cells

Adaptive immunity, especially CD8+ cytotoxic T cells, plays a key role in antitumor immunity and the effectiveness of ICIs. An imbalance in adaptive immunity within the PDAC TME mainly appears as serious issues with antigen presentation, functional exhaustion, and low numbers of effector T cells, and increased infiltration and activation of immunosuppressive cells. Overall, these factors contribute to the failure of antitumor immune responses.

Because of its low mutation burden, PDAC exhibits a paucity of high-quality neoantigens. However, one study demonstrates that introducing a single potent neoantigen can reverse the immune “cold” state and activate T-cell-mediated clearance of tumor responses (85). This suggests that neoantigens play a crucial role in initiating immunotherapy. Later, in identifying unique neoantigens specific to long-term pancreatic cancer survivors, tumor-infiltrating T cells from LTS patients were found to cross-react with both cancer neoantigens and similar non-cancer microbial antigens (86). I In a pancreatic cancer mouse model, mice that received fecal microbiota transplantation from LST patients showed a significant increase in the number of CD8^+^ T cells and activated T cells (CD8^+^/IFNg^+^ T cells) (49). Further research indicates that specific microorganisms inhabiting the gut or tumors can produce peptide segments that structurally resemble pancreatic cancer tumor neoantigens. These microbial peptides can be internalized by antigen-presenting cells (APCs) and cross-presented to CD8^+^ T cells through MHC class I molecules, thereby triggering cytotoxic responses against the tumor cells (87). The study also suggests that a highly diverse microbiome correlates with better responses to immunotherapy and higher survival rates, opening new possibilities for improving immunotherapy through microbial means modulation (87).

Recently, a new gut bacterium, *YB328*, isolated from the feces of responders to immunotherapy, was found to promote tumor-infiltrating PD-1^+^ CD8^+^ T cells with a more diverse T cell receptor repertoire (88). This suggests that these cells can recognize a broader range of tumor antigens, thereby preventing rapid loss of individual T cell clones and improving the effectiveness of PD-1 blockade therapy. Although this mechanism has not been directly validated in pancreatic cancer, it provides novel insights for future studies on training effector T cells. This should be based on identifying unique microbiomes in immune responders to enhance immunotherapy. A recent study using a pancreatic cancer mouse model revealed that microbial distribution in pancreatic tumor tissues exhibits T cell-associated spatial patterns heterogeneity (89). Tumors enriched in CD8^+^ T cells showed genus-level enrichment of *Arthrobacter* and *Bacillus*, as well as species-level enrichment of *Methylobacteriaceae*. Conversely, tumors lacking CD8^+^ T cells showed an increase in *Salirhabdus* at the genus level and a specific enrichment of certain species of *Oxalobacteraceae (*89). This finding provides particular directions for microbial research in studies aimed at enhancing the efficacy of immunotherapy through microbiota-mediated modulation.

### 3.3 Metabolites indirectly regulate immunity: classification and implications for immunotherapy

Microbial metabolites, acting as small-molecule messengers, can diffuse from the gut to the systemic circulation, where they modulate antitumor immunity and constitute a crucial component of the TME.

#### 3.3.1 Short-chain fatty acids

In short-chain fatty acids (SCFAs), butyrate and its associated microbiota were found to be significantly decreased in fecal and tumor tissue samples from pancreatic cancer patients (50). Meanwhile, the bacterial genera enriched in tumors of LTS patients possess substantial capacity to produce SCFAs, including butyrate, valeric acid, and isovaleric acid (90). Preclinical studies suggest that supplementation with specific SCFAs may exhibit antitumor effects. However, clinical data in patients with PDAC remain lacking. Mouse models of melanoma and pancreatic cancer showed that certain SCFAs can significantly boost the antitumor capacity of CD8+ T cells through a combined metabolic-epigenetic programming mechanism (91). Specifically, the HDAC class I inhibitory activity of valerate and butyrate increases the expression of TNF-α and IFN-γ effector molecules in cytotoxic T lymphocytes (CTLs) (91). The HDAC class I inhibitory effect was mediated by pentanoate- and butyrate-producing *M. massiliensis* and *M. elsdenii*, as well as by the butyrate-producing *F. prausnitzii* and *A. hadrus (*91). Butyrate activates the mTOR signaling pathway, prompting T cells to adopt a glycolytic metabolic mode. This supplies the essential energy and raw materials necessary for rapid T cell proliferation, cytokine production, and sustained survival within the body (91). Additionally, in PDAC mouse models, butyrate can remodel the TME by reducing collagen deposition, decreasing myofibroblast infiltration, lowering proangiogenic factor production, and inhibiting M2-type TAMs polarization (92). This reverses the immunosuppressive TME and enhances the delivery of chemotherapy drugs. ROR1-specific CAR T cells from humans and mice treated with valerate and butyrate exhibit significant antitumor activity, further underscoring the clinical potential of pretreated CTLs and a supplemental SCFA-producing microbiota for synergistic adoptive cell therapy (91). However, the optimal timing, dosage, and route of administration for SCFAs remain unclear, and their effects mediated by the gut microbiota may vary among individuals, creating significant challenges for precision interventions.

#### 3.3.2 Polyamines and L-arginine

Unlike the decline in relevant SCFAs, the levels of microbially derived polyamines in the cystic fluid of patients with PDAC were significantly elevated (93). This promotes tumor cell growth and stimulates M2 macrophage polarization, thereby enhancing immune evasion (93). In genetically engineered PDAC mouse models (KPC), the synchronized increase in *Lactobacillus reuteri* and polyamine concentrations indicates a potential role for *Lactobacillus reuteri* in polyamine production (94). Existing approaches to blocking polyamines primarily focus on enzyme- and transporter-inhibitor pathways, whereas microbial-related pathways remain underdeveloped. Interestingly, arginine, a key precursor in polyamine biosynthesis, has recently been found to be depleted in the pancreatic cancer TME (95). Pancreatic cancer cells can acquire polyamines via the ornithine transaminase pathway (95). Canale’s team increased tumor-specific levels of L-arginine, a crucial precursor in polyamine biosynthesis, using engineered probiotics (96). In a mouse model of colon cancer, this approach increased the number of tumor-infiltrating T cells while reducing the proportion of Treg cells, thereby boosting the antitumor response to ICIs (96). Therefore, targeting the microbe-polyamine axis or regulating the metabolism of the polyamine precursor arginine with engineered probiotics offers a new translational strategy to overcome immune suppression in pancreatic cancer.

#### 3.3.3 Bile acids

Furthermore, bile acids (BAs) are believed to be associated with many risk factors for pancreatic cancer. They can serve as important signaling molecules that regulate disease development and progression (97). Unlike Joshi’s finding that both deoxycholic acid (DCA) and chenodeoxycholic acid promote malignant progression in pancreatic cancer cells by activating the FXR receptor and upregulating the oncogene MUC4 through the Src/FAK/c-Jun signaling pathway axis (98). *In vitro*, high concentrations of BAs mixtures were found to inhibit cancer cell migration and promote apoptosis by inducing ROS production and suppressing the epithelial-mesenchymal transition (EMT) process (99). Primary BAs are metabolized by intestinal microbiota in the duodenum into secondary BAs (100). Different intestinal microbiota produce secondary BAs with distinct modifications, leading to their diversity (101). Other types of BAs induce, in a dose-dependent manner, the cancer-associated genes MUC4 and COX-2, thereby promoting tumor growth (102, 103). DCA, as a key stimulatory bile acid in PDAC, is recognized for its dual properties: it exhibits antitumor characteristics by inhibiting epithelial-mesenchymal transition, reducing tumor stem cells, and inducing oxidative/nitrosative stress. At the same time, it promotes tumor growth by inducing a hypermetabolic state that generates chemoresistance (104). In contrast, Kim et al. reported that another secondary bile acid, ursodeoxycholic acid, effectively inhibits EMT and cancer stem cell formation by decreasing ROS levels and by increasing superoxide dismutase 2 (105). This duality strongly indicates that the gut microbiota, through its metabolic activity in regulating bile acid profiles, may serve as a key environmental factor affecting the biological behavior of pancreatic cancer. However, the role of deoxycholic acid varies considerably across different cancer contexts, showing high environmental influence dependence (106). The mechanism of action of specific secondary BAs in the body environment requires further investigation.

#### 3.3.4 Tryptophan metabolite

In addition to the classic metabolites mentioned earlier, studies have shown that *Lactobacillus metabolizes* dietary tryptophan into indole, which activates the aryl hydrocarbon receptor (AhR) in macrophages, promoting an immunosuppressive phenotype and reducing inflammatory T-cell infiltration (107). Tintelnot found that, in a humanized gnotobiotic mouse model of PDAC, combining the microbial-derived tryptophan metabolite indole-3-acetic acid with chemotherapy reduced tumor growth via a mechanism independent of AhR (108). Although 3−IAA shows strong preclinical synergy with FOLFIRINOX/GnP, clinical translation remains to be defined.

#### 3.3.5 Trimethylamine N-oxide

Trimethylamine N-oxide (TMAO) is a metabolite mainly produced by gut microbiota. In an *in situ* PDAC mouse model, TMAO is believed to directly enhance the immune-stimulatory phenotype of TAMs, strengthen the antitumor immune response of effector T cells, reduce tumor burden, and increase their responsiveness to ICIs (109). However, TMAO is also associated with an increased risk of cardiovascular events, and its safety in cancer treatment requires further validation (110).

### 3.4 Driving the development of treatment resistance

Multiple small-scale clinical trials have shown that monotherapy or combination immunotherapy for pancreatic cancer has not produced satisfactory results (10, 111, 112). However, combining immunotherapy with radiotherapy and chemotherapy is thought to improve efficacy (113). Along with the aforementioned role of microorganisms in shaping the immune TME, a 2017 study revealed that *Gammaproteobacteria* within tumors can metabolize the chemotherapy drug gemcitabine to an inactive form by producing the bacterial enzyme cytidine deaminase (CDD_L_), and antibiotics targeting these bacteria can help prevent this resistance (38). Photodynamic antimicrobial therapy targeting *Gammaproteobacteria* effectively inhibited gemcitabine metabolism and improved chemotherapy effectiveness (114). Meanwhile, oral administration of multiple probiotics can enhance the effectiveness of gemcitabine chemotherapy and improve tolerance to chemotherapy (115–117).

A study on the longitudinal changes in the gut microbiome of melanoma patients after immunotherapy indicated that *Eisenbergiella* sp.*, Bacteroides wexlerae, Clostridium* sp*iroforme*, and *Erysipelotrichaceae* are linked to resistance to ICIs. Similar research should be done in patients with pancreatic cancer undergoing immunotherapy or combination therapy to identify the microbial communities associated with resistance, aiding targeted elimination and reducing the adverse effects of resistance (116).

## 4 Microbial control methods

**Figure 2:**
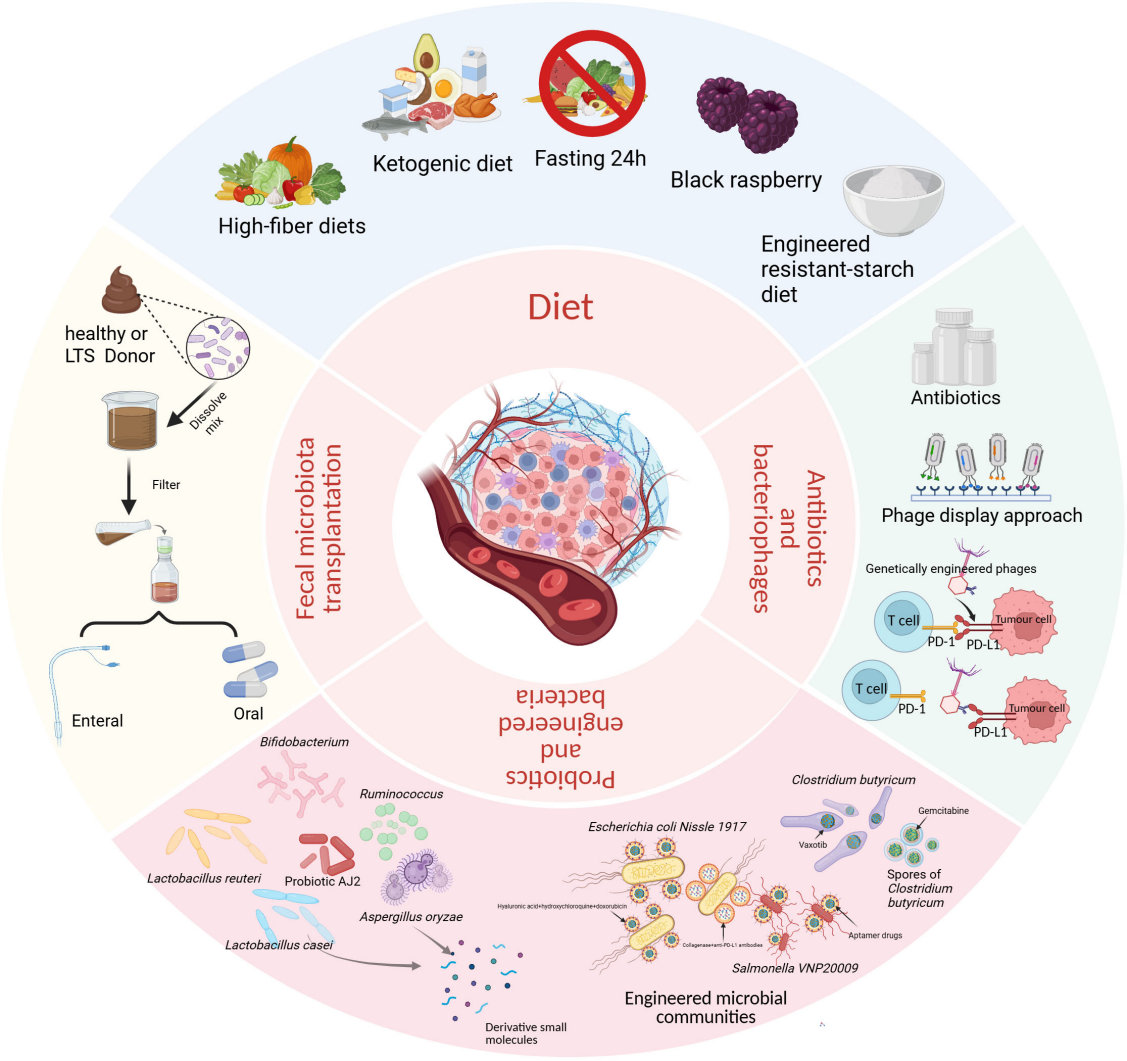
Core Intervention Methods for Microbial Regulation. Diet: fasting 24h, ketogenic diets, high-fiber diets, black raspberry, engineered resistant-starch diet. Fecal Microbiota Transplantation: TME from healthy or LTS patients to recipients to restore gut microbial balance. Antibiotics and Phages: Targeted elimination of tumor-promoting or drug-resistant microbial populations, genetically engineered bacteriophages to block the PD-1/PD-L1 Axis. Probiotics and Engineered Microorganisms: Supplementing probiotics or their metabolites directly modulates the TME. Engineered microorganisms (such as *Escherichia coli Nissle 1917*, *Salmonella VNP20009*, *Clostridium butyricum*, and its spores) are engineered to target specific substrates or deliver drugs. Created in BioRender. Yuan, Y. (2026) https://BioRender.com/395il9b.

The microbiome influences host antitumor immunity through the mechanisms mentioned earlier and potentially other pathways, thereby affecting the clinical response and outcomes of patients undergoing cancer immunotherapy. It might also be possible to predict the effectiveness of immunotherapy. Specific interventions that modify the microbiome could lead to the development of new therapeutic strategies as valuable adjuncts to current antitumor treatments and immunotherapies (**Figure 2**).

### 4.1 Diet

Dietary intake, as an important environmental factor, is believed to influence tumor metabolism and thereby enhance treatment effectiveness (118). Gemcitabine-treated pancreatic cancer xenograft mouse models that underwent 24-hour fasting before treatment exhibited significantly slower tumor growth. Both *in vivo* and *in vitro* assays confirmed that fasting enhances gemcitabine uptake and counteracts chemoresistance caused by mTOR activation (119). Similarly, the ketogenic diet (extremely low in carbohydrates, low in protein, high in fat) shows significant potential to limit tumor growth in KPC mouse models when combined with standard PDAC treatments, chemotherapeutics (120). A related randomized clinical trial investigating ketogenic diets combined with standard chemotherapy in metastatic pancreatic cancer patients is currently underway (NCT04631445). A retrospective review, based primarily on multiple mouse models and supplemented by limited rat models and human studies, concludes that high-fat diets typically decrease *Bacteroidetes* and increase *Firmicutes* and *Proteobacteria*. These changes promote inflammation and increased permeability, thereby allowing bacteria and their metabolites to translocate and potentially exacerbate microbial dysbiosis in pancreatic cancer (121). High-fiber diets may enhance treatment effectiveness by promoting the growth of beneficial microbiota, such as *Bifidobacterium* and *Lactobacillus*, which produce helpful metabolites such as butyrate (122). Similarly, feeding pancreatic cancer xenograft mice an engineered diet replacing corn starch with resistant starch for 18 days significantly slowed tumor growth. This antiproliferative effect may be linked to reduced pro-inflammatory gut microbes and an increase in butyrate-producing *Lachnospiraceae* (123). A 5% black raspberry diet fed for six weeks modulated immune activity in both xenograft and genetically engineered PDAC mouse models by increasing CD8^+^T cell and NK cell infiltration while reducing MDSCs—an effect similarly linked to diet-induced butyrate production (124).

### 4.2 Fecal microbiota transplantation

Due to the difficulty in identifying specific species within the gut microbiota and the potential impact of supplementing with a single bacterial strain on the gut microbiota diversity (125). FMT is considered the simplest method for directly changing gut microbiota composition. It is believed to have the potential to restore immune balance, reduce immune-related adverse events, and improve immunotherapy effectiveness (126). Some clinical studies have shown that combining FMT and immunotherapy has demonstrated significant efficacy in melanoma, colorectal cancer, and other gastrointestinal solid tumors (127–129). In preclinical pancreatic cancer studies, the donor source of FMT, LTS versus STS, directly determines its regulatory effects on the TME. Foundational work by the Riquelme group provides direct evidence supporting this idea. They transplanted gut microbiota from pancreatic cancer LTS and STS donors into syngeneic pancreatic cancer mouse models, respectively. Results showed that mice receiving LTS-derived FMT exhibited favorable changes in intratumoral microbiome composition, along with increased CD8^+^ T-cell activity (49). Meanwhile, the accumulation of MDSCs and Tregs was suppressed, leading to a significant slowdown in tumor progression. In contrast, STS-derived microbiota failed to induce such beneficial immune reprogramming. Instead, they likely exacerbated immunosuppression by increasing TME fibrosis and promoting M2-type TAMs polarization, thereby impairing the efficacy of ICIs (49). This critical difference underscores the need for rigorous donor selection and functional characterization in FMT-based interventions. Currently, Phase I clinical trials are investigating the effect of FMT in pancreatic cancer are underway to assess the safety and effectiveness, as well as the safety of combining it with chemotherapy. (NCT 06393400 and NCT 04975217).

### 4.3 Antibiotics and phages

The impact of antibiotics on immunotherapy is twofold. On the one hand, antibiotics help eliminate drug-resistant microorganisms associated with chemotherapy and immunotherapy, thereby improving treatment effectiveness. For precise regulation, clarifying their distinct mechanisms and exploring more targeted alternative strategies is critical.

#### 4.3.1 Antibiotics in reversing microbiome-mediated drug resistance

Geller et al.’s groundbreaking work showed that intratumoral *Gammaproteobacteria* metabolize the chemotherapeutic drug gemcitabine into its inactive form through bacterial cytidine deaminase expression, leading to treatment failure (38). Eradicating these bacteria with the targeted antibiotic ciprofloxacin reverses this resistance and restores gemcitabine efficacy (38). Furthermore, metagenomic data analysis of pancreatic cancer patient samples suggests that antibiotic resistance genes carried by gut-derived bacteria, such as *Klebsiella pneumoniae*, *Klebsiella oxytoca*, and *Acinetobacter mucosus*, may reduce tumor cell chemosensitivity to drugs such as gemcitabine through mechanisms including increased efflux pump activity and drug-target modification (130). Findings from these preclinical models confirm that bacteria not only directly degrade drugs but also contribute to chemoresistance by enhancing the intrinsic tolerance of tumor cells or TME. Subsequent studies developed photodynamic antimicrobial therapy targeting such drug-resistant bacteria, which effectively inhibits bacterial gemcitabine metabolism and enhances chemotherapeutic efficacy (114). Collectively, these mechanisms highlight the value of antibiotics to overcome both direct and indirect bacterial-mediated chemoresistance.

#### 4.3.2 Antibiotics and ICIs response: a double-edged sword

Beyond chemoresistance, the impact of antibiotics extends to the emerging realm of immunotherapy for PDAC. While the clinical success of ICIs in PDAC has been limited, combinatorial strategies with chemotherapy are actively being explored to overcome the immunosuppressive TME (131, 132). In this context, the impact of antibiotics used for infection management in cancer patients on the efficacy of ICIs has also become a critical clinical consideration. Multiple observational studies have reported that administering broad-spectrum antibiotics before or after ICIs impairs the effectiveness of immunotherapy and reduces patient survival (133–135). The underlying mechanism involves the indiscriminate disruption of gut microbiota diversity and homeostasis by these antibiotics. This process depletes beneficial commensal bacteria, such as *Akkermansia muciniphila*, *Alistipes* spp., *Ruminococcus* spp., *Eubacterium* spp., and *Lactobacillus johnsonii*, which are critical for initiating and maintaining robust antitumor T cell responses (133, 134). Such dysbiosis shifts the TME toward an immunosuppressive state, for example, by reducing tumor-infiltrating CD8^+^ T cells and increasing regulatory T cells, thereby abrogating ICIs activity.

To address this issue, narrow-spectrum antibiotics and microbiota-protective strategies have become important research focuses. These methods involve screening for specific pathogenic or drug-resistant strains through metagenomic sequencing and developing targeted antibiotic combinations to maintain beneficial microbiota. Alternatively, co-administering antibiotics with probiotics, prebiotics, or FMT can help protect or quickly restore the gut ecosystem necessary for optimal immunotherapy efficacy.

#### 4.3.3 Phages

Phages, as a complementary therapy for antibiotic resistance, exhibit both specificity and immune-modulatory abilities (136), and have been reported to be effective in treating pancreatitis caused by multidrug-resistant *Acinetobacter baumannii (*137). Phage display technology identified plectin-1 as a novel PDAC biomarker in 2008, offering new molecular and imaging tools for pancreatic cancer diagnosis (138). In melanoma, genetically engineered filamentous phages are considered cost-effective and highly effective tumor-targeted immunotherapy agents that inhibit tumor cell immune evasion by blocking the PD-1/PD-L1 axis (139). The bidirectional effects of antibiotics on antitumor therapy highlight the importance of accurately targeting microorganisms associated with cancer. Phages offer an economically feasible targeting approach, demonstrating the potential for precise modulation of the microbiome within pancreatic tumors.

### 4.4 Probiotics and engineered bacteria

Probiotics may act as a natural adjunct approach for pancreatic cancer immunotherapy by regulating gut microbiota balance, improving intestinal barrier function, and modulating immune cell activity. Building on this foundation, genetic engineering techniques further enhance microbial targeting and functional specificity, turning engineered bacteria into innovative tools to precisely breach the TME barrier and potentially synergize with immunotherapy.

#### 4.4.1 Probiotics: natural microbiota regulation and immune synergy

Probiotics are defined as live microorganisms that confer health benefits on the host when administered in adequate amounts (140). Common probiotics include *Bifidobacterium*, *Lactobacillus*, *Acinetobacter mucosus*, and *Ruminococcus*. Specific probiotic strains have been shown in preclinical studies to modulate immune cell function in the TME. For example, *Bifidobacterium* and *Ruminococcus* were associated with enhanced dendritic cell activity and increased CD8^+^ T cell infiltration into tumors, which may contribute to improved efficacy of anti-PD-L1/PD-1 therapy in those models (141, 142). In a pancreatic cancer mouse model, dietary supplementation with *Lactobacillus reuteri* was associated with increased NK cell infiltration into the TME and correlated with enhanced antitumor activity (84). Similarly, pancreatic cancer models fed with the probiotic strain *AJ2* was associated with restored NK cell cytotoxicity and IFN-γ secretion (143). Beyond modulating immune cell function, a probiotic mixture dominated by *Bifidobacterium* and *Lactobacillus* species was reported to delay pancreatic epithelial-mesenchymal transition and reduce chemotherapy-related toxicity in a xenograft model (117).

Probiotics can produce systemically active antitumor molecules through intestinal fermentation. For instance, *Aspergillus oryzae* and its metabolite heptelidic acid activate the p38 MAPK pathway, whereas ferrichrome from *Lactobacillus casei* induces endoplasmic reticulum stress; both pathways have been shown to induce apoptosis in pancreatic cancer cells (144, 145). Furthermore, ferrichrome has been shown to activate TLR4 signaling in mouse models, leading to M1 polarization of TAMs, increased CD8^+^ T cell infiltration, and improved response to anti-PD-L1 therapy (146).

In addition to providing probiotics, prebiotics, and nondigestible food ingredients that serve as nourishment for beneficial bacteria in your gut, they also serve as beneficial substrates that support probiotic colonization. These may indirectly affect treatment results and aid in repairing damaged intestinal barriers (147). In the context of PDAC, preclinical evidence suggests that dietary fibers can increase the abundance of SCFA-producing bacteria, which may indirectly modulate antitumor immunity (122). However, direct evidence linking specific prebiotic supplementation to improved immunotherapy outcomes in PDAC patients is currently lacking. Synbiotics, which combine probiotics and prebiotics, have been shown in a randomized controlled trial to enhance anti-tumor immune cell infiltration and lead to fewer surgical complications compared to the use of probiotics alone (148). This highlights a potential combinatorial approach, though larger confirmatory studies are needed.

Despite promising preclinical data, several challenges must be addressed for clinical translation. The optimal probiotic strain, dosage, timing, and duration for PDAC remain undefined and likely vary among individuals due to baseline microbiome differences. Safety concerns, though generally low for traditional probiotics, include the risk of bacteremia in immunocompromised patients (149). Furthermore, the regulatory pathway for probiotics as cancer therapeutics is complex, as they are often classified as dietary supplements rather than drugs, which limits the requirement for rigorous clinical trial validation of specific health claims (150).

#### 4.4.2 Engineering bacteria: precision targeting and functional enhancement

Genetic engineering can improve microorganisms with abilities such as targeted delivery, tissue penetration, and combination drug release. These modified microbes are designed to overcome key barriers in the TME, including stromal fibrosis, immune suppression, and poor drug infiltration, and with the aim of synergizing with immunotherapy.

Engineered facultative anaerobic bacteria can target hypoxic tumor regions and degrade dense stroma locally. A key example is *Escherichia coli Nissle 1917* (ECN), which can be loaded with collagenase or hyaluronidase to disrupt the matrix barrier, thereby enhancing the penetration and accumulation of chemotherapeutics and ICIs, such as anti-PD-L1 antibodies (151, 152). Similarly, *Clostridium butyricum* has been modified to deliver the TGF-β inhibitor Vaxotib, thereby suppressing extracellular matrix production (153). This promotes drug and immune cell infiltration while competitively inhibiting pro-tumor γ-proteobacteria, thus modulating the tumor microbiome (153). While these strategies effectively remodeled the TME in mouse models, their safety regarding non-targeted tissue damage and systemic immune responses in humans remains to be evaluated.

In addition to targeting the TME, engineered bacteria in the following preclinical models can also serve as carriers for chemotherapeutic drugs and immunomodulators, allowing precise and sustained drug delivery to tumors. For example, ECN loaded with anti-PD-L1 antibodies, autophagy inhibitors, and chemotherapeutics reduces immunosuppression and increases intratumoral drug levels (151, 152). Similarly, *Salmonella VNP20009* engineered to deliver aptamer drugs has been shown to foster an immune-activated TME, suggesting potential for enhancing both chemotherapy and immunotherapy (154). Additionally, a harsh-condition-tolerant spore of *Clostridium butyricum* has been developed for the delivery of gemcitabine; after oral administration, it targets pancreatic tumors specifically via the gut-pancreatic axis (115).

## 5 Methodological limitations and future directions

### 5.1 Low biomass and pollution control

Pancreatic tissue is a low-microbial-biomass environment, making it susceptible to exogenous microbial DNA contamination from reagents, laboratory settings, and sampling procedures, which may produce false-positive signals (155). Improper handling and storage can degrade microbial DNA, reducing signal intensity and increasing the risk of false negatives (155). Batch effects arising from different platforms and data collection methods also require consideration (156). Although negative controls and bioinformatic filtering can partially address contamination and batch effects, future studies should implement more rigorous experimental controls and standardized decontamination protocols. Recent consensus reports outline strategies to reduce contamination and cross-contamination, and establish minimum reporting standards for contamination-related information and decontamination steps (157).

### 5.2 Correlation and causation

Most human studies show links between microbial features and clinical outcomes. However, baseline gut microbiota can differ based on patient location, diet, and smoking/alcohol habits, leading to variations in tumor-related microbial profile shifts (50, 158). Treatments also change microbial composition; for example, in tumor−bearing rats with experimental cancers, irinotecan increased *Clostridium cluster XI* and *Enterobacteriaceae* in tumor-bearing rats (159, 160). Establishing large-scale, multicenter databases with detailed clinical metadata is therefore essential for validating biomarkers and supporting clinical translation. Furthermore, tumors exhibit spatial heterogeneity, potentially harboring distinct microbial communities in different regions, such as immune-cell-deficient versus immune-cell-enriched areas (89). Bulk sequencing masks this micro-geographic distribution. Future mapping of microbial–host interactions will benefit from combining single-cell and spatial transcriptomics with microbial detection (17).

Current causal inference primarily relies on animal models, such as xenograft, germ-free, and humanized FMT models. Xenograft models, which involve implanting human tumor tissue into immunodeficient mice, are useful for evaluating drug responses but cannot fully reproduce the entire immune–microbiome system interactions (161, 162). Germ-free models represent a non-physiological extreme and prevent the study of inter-microbial ecology, such as competition or cooperation. Although mono-colonization studies can demonstrate causality, they do not capture the complexity of human microbial communities ecosystems (29). Humanized FMT models depend on donor, recipient, and procedural variables and therefore require standardized protocols to ensure reproducibility. Patient−derived organoids, which retain key TME features, provide a complementary platform (163). Integrating organoids with animal models may provide a more comprehensive research strategy (164).

### 5.3 Clinical translation challenges

Although FMT is approved for recurrent *Clostridioides difficile* infection (165), its long-term efficacy and safety in non−infectious diseases require further validation. FMT outcomes can vary based on donor and recipient microbiome characteristics, as well as differing clinical regulations, drug policies, and medical practices globally (166). Establishing international consensus on donor screening and production protocols is therefore necessary to improve safety and generalizability (167).

Engineered microbial therapies, such as modified bacteria and phages, provide high versatility and represent a promising approach in microbiome-based precision medicine (168). While investigating different disease models, they must address biosafety and ethical concerns, such as preventing unintended genetic recombination or the spread of antibiotic resistance (169). The variety of these artificial therapies complicates regulatory standardization, highlighting the need for validated preclinical models and mutually agreed-upon regulatory frameworks (170).

## 6 Summary and outlook

This review discusses the role of the gut and tumor microbiome in pancreatic cancer metastasis and treatment response. Evidence indicates that the microbiome actively influences the immunosuppressive TME through cellular components, metabolites, and immune interactions, thereby impacting disease progression and therapy. This provides a strong rationale for targeting the microbiome to enhance immunotherapy. However, translating these findings into clinical practice still faces significant challenges.

Microorganisms often participate in migration and colonization collectively. In addition to their interactions with immune cells, the patterns of their internal interactions also deserve attention. Studies have shown that the microbial interaction patterns in pancreatic cancer patients are less complex than those in healthy individuals, with reduced tightness (171). Such changes in interaction patterns may also influence the development of the pancreatic cancer TME. Future research should focus on the overall structure of microbial communities, the stability of their interaction networks, and their collective metabolic functions. A functional ecological niche may be more significant than the presence of specific taxa. Integrating metagenomics, metabolomics, and single-cell sequencing will help decipher this complex ecosystem.

Current analyses linking tumor-associated microbes to prognosis yield conflicting results, underscoring significant inter-patient microbiome diversity as a key obstacle to personalized therapy. This variation arises from host genetics, diet, medication history, tumor molecular subtype, and anatomical location. Future clinical trials should therefore consider these factors and develop microbiota-based patient stratification to identify those most likely to benefit from specific treatments. All microbiome-targeting strategies must be validated for effectiveness and safety through carefully designed randomized controlled trials, with close monitoring of potential effects on immunotherapy-related adverse events.

In summary, microbiome research is transforming our understanding of PDAC. Through interdisciplinary collaboration and technological innovation, targeting the microbiome is expected to become a key part of multimodal PDAC therapy, offering a new strategy to improve outcomes for this immunologically cold tumor.
